# Screening of 71 *P. multocida* Proteins for Protective Efficacy in a Fowl Cholera Infection Model and Characterization of the Protective Antigen PlpE

**DOI:** 10.1371/journal.pone.0039973

**Published:** 2012-07-05

**Authors:** Tamás Hatfaludi, Keith Al-Hasani, Lan Gong, John D. Boyce, Mark Ford, Ian W. Wilkie, Noelene Quinsey, Michelle A. Dunstone, David E. Hoke, Ben Adler

**Affiliations:** 1 Australian Research Council Centre of Excellence in Structural and Functional Microbial Genomics, Monash University, Clayton, Victoria, Australia; 2 Department of Biochemistry and Molecular Biology, Monash University, Clayton, Victoria, Australia; 3 Department of Microbiology, Monash University, Clayton, Victoria, Australia; 4 CSIRO Livestock Industries, Australian Animal Health Laboratory, Geelong, Victoria, Australia; 5 Veterinary Pathology and Anatomy, University of Queensland, Brisbane, Queensland, Australia; Instituto Butantan, Brazil

## Abstract

**Background:**

There is a strong need for a recombinant subunit vaccine against fowl cholera. We used a reverse vaccinology approach to identify putative secreted or cell surface associated *P. multocida* proteins that may represent potential vaccine candidate antigens.

**Principal Findings:**

A high-throughput cloning and expression protocol was used to express and purify 71 recombinant proteins for vaccine trials. Of the 71 proteins tested, only one, PlpE in denatured insoluble form, protected chickens against fowl cholera challenge. PlpE also elicited comparable levels of protection in mice. PlpE was localized by immunofluorescence to the bacterial cell surface, consistent with its ability to elicit a protective immune response. To explore the role of PlpE during infection and immunity, a *plpE* mutant was generated. The *plpE* mutant strain retained full virulence for mice.

**Conclusion:**

These studies show that PlpE is a surface exposed protein and was the only protein of 71 tested that was able to elicit a protective immune response. However, PlpE is not an essential virulence factor. This is the first report of a denatured recombinant protein stimulating protection against fowl cholera.

## Introduction

Bacterial vaccines can be divided into three main categories: killed whole cell vaccines (bacterins), live attenuated vaccines and native or recombinant subunit vaccines. Subunit vaccines offer the advantage of using a defined component of the infectious microorganism to stimulate a protective immune response and, since subunit vaccines cannot replicate in the host, there is no risk of reversion to virulence. Furthermore, subunit vaccines have a number of other benefits including their defined composition, simplified large-scale production and the ability to make specific modifications and improvements to the proteins [1]. However, identification of single protective antigens is very difficult. Recently, the advent of whole genome sequencing techniques has allowed the development of a rapid and comprehensive method for antigen discovery, termed reverse vaccinology. The concept of reverse vaccinology, first applied to *Neisseria meningitidis* [2], has now been tested in three additional organisms [3]–[5]. This method uses bioinformatics analysis of genomes to predict open reading frames (ORFs) encoding cell-surface or secreted proteins. These ORFs are then expressed in a heterologous expression system and tested for their ability to elicit a protective immune response in animal models of infection. Cumulatively, these works have expressed more than 800 proteins with 17 protective antigens identified; representing an identification rate of between 1 and 5%.

*Pasteurella multocida* is a Gram-negative bacterium that is the causative agent of numerous diseases, including hemorrhagic septicemia in ungulates, fowl cholera in birds, atrophic rhinitis in swine, pneumonia and shipping fever in cattle, and snuffles in rabbits. The bacterium can also cause infections in humans, often following dog or cat bites [6]. *P. multocida* strains can be differentiated serologically into five distinct capsular groups, designated A, B, D, E and F [7], and 16 lipopolysaccharide (LPS) types, designated 1 to 16 [8]. While immunity is dependent predominantly on LPS types, genome analysis has revealed that these serological classifications are only represented by minor differences at the genome level.

At present commercial vaccines are available for a range of diseases caused by *P. multocida* [9]. Bacterins are widely used to vaccinate against fowl cholera, hemorrhagic septicemia, and atrophic rhinitis [10]–[12]. However, bacterins suffer from variable efficacy, a failure to elicit immunity against heterologous LPS serotypes and a short duration of immunity. Commercial live attenuated vaccines are also available for fowl cholera [13] and live vaccines for hemorrhagic septicemia have been tested [14], . However, the basis of attenuation for these strains is unknown and the vaccines have occasionally reverted to virulence and caused disease outbreaks [13]. Defined attenuated strains capable of stimulating protective immunity have been constructed [10], [16], [17] and one has recently been licensed for use in Australia [18], [19].

We have reported an extensive set of outer membrane (OM) and OM-associated proteins through bioinformatics analysis of the *P. multocida* genome [20], [21]. Due to their predicted localization as secreted or surface exposed proteins, they were proposed to have the potential to elicit a protective immune response. Thus, the aim of this study was to assess vaccine candidates through a reverse vaccinology approach. Accordingly, 71 of these proteins were tested in recombinant form for immunogenicity and for their ability to protect against lethal *P. multocida* infection. Only a single recombinant protein was capable of eliciting a protective immune response.

## Results

### Selection, Expression and Purification of Vaccine Target Antigens

Previous bioinformatics analysis of the *P. multocida* Pm70 genome sequence identified genes encoding proteins with vaccine potential [20]. Potential protective antigens were defined as surface exposed or secreted proteins and those with similarity to proteins with putative or confirmed roles in infection and/or immunity. One hundred and five candidate genes were identified from this analysis. Each of these candidate genes was cloned into expression vectors and, based on the expression characteristics of the recombinant plasmids, 71 proteins were selected for testing as they could be purified in sufficient quantities for vaccination experiments. The 71 genes encoding these proteins were cloned into a range of expression vectors and the clones with the best expression characteristics selected (Table 1). Four proteins were expressed and purified from the pBAD-DEST49 Gateway® destination vector that features a thioredoxin fusion tag. A modified Gateway-adapted expression vector containing a NusA solubility tag (pDEST-41BA) was selected for expression of 29 proteins; nine of these proteins were expressed and purified in soluble form. Another 40 proteins (of which three were soluble) were expressed from pDEST-17 and purified as Hisx6-tagged proteins.

**Table 1:** Recombinant *P. multocida* proteins tested as vaccine candidates in chickens.

Gene number a	Predicted molecular mass	Protein name and predicted function	Expression vector b	Solubility c	Signal P d	Immunogenic e	Reaction with *P.m* f	Protection
controls g		Killed whole cells						**+**
		Adjuvant only						−
PM0040	81.2	PfhR - putative outer membrane hemin receptor	pDEST-17	I	+	+	−	−
PM0055	28.5	acyl-CoA thioester hydrolase YfbB	pDEST-41BA	I	−	+	−	−
PM0056	54	FhaC – Filamentous haemagglutinin two partner secretion protein	pDEST-17	I	−	+	−	−
PM0076	74.5	MapC protein	pDEST-17	I	+	+	−	−
PM0098	41.9	OapA - Cell envelope opacity-associated protein A	pBAD-DEST49	I	−	+	−	−
PM0243	59.5	Pm0243 - hypothetical protein	pDEST-41BA	S	+	+	+	−
PM0246	24.3	LolB - outer membrane lipoprotein	pDEST-17	I	+	+	−	−
PM0300	109.6	HgbA - TonB-dependent haemoglobin receptor	pDEST-17	I	+	+	−	−
PM0331	21.7	OmpW - outer membrane protein	pDEST-17	I	+	+	−	−
PM0337	113.2	HgbB - Haemoglobin/haptoglobin binding protein	pDEST-17	I	+	+	−	−
PM0355	29.9	Pm0355 - hypothetical protein	pDEST-17	I	−	+	−	−
PM0368	26.9	Pm0368 - hypothetical protein	pDEST-41BA	S	+	+	−	−
PM0388	37.3	OmpH1 - outer membrane porin	pDEST-17	I	+	+	+	−
PM0389	38.6	OmpH2 - outer membrane porin	pDEST-17	I	+	+	−	−
PM0442	23.7	Pm0442 - immunogenic membrane protein	pDEST-41BA	S	+	+	+	−
PM0519	12.5	Pm0519 - hypothetical protein	pDEST-17	I	−	+	−	−
PM0527	50.6	TolC1 - outer membrane efflux channel	pDEST-17	I	+	+	+	−
PM0542	75.1	Glgx - glycogen operon protein	pDEST-41BA	I	−	+	−	−
PM0554	15.5	PCP - peptidoglycan-associated lipoprotein cross reacting protein	pDEST-41BA	I	+	+	+	−
PM0586	29.9	Plp4- lipoprotein	pDEST-17	I	+	+	−	−
PM0612	11.4	Pm0612 - Hypothetical outer membrane protein	pDEST-41BA	I	−	+	−	−
PM0618	99.4	Pm0618 - aminopeptidase N	pDEST-41BA	I	−	+	−	−
PM0659	214.3	Pm0659 - Large extracellular alpha-helical protein, lipoprotein	pDEST-17	S	+	+	−	−
PM0680	77.4	PrlC - oligopeptidase A	pDEST-41BA	I	−	+	+	−
PM0708	20.4	Pm0708 - hypothetical outer membrane lipoprotein	pDEST-17	I	+	+	−	−
PM0714-1 h	91.4	Hsf - hypothetical outer membrane protein	pDEST-17	I	−	+	−	−
PM0714-2 h	93.7	Hsf - hypothetical outer membrane protein	pDEST-41BA	I	−	+	−	−
PM0714-3 h	94.9	Hsf - hypothetical outer membrane protein	pDEST-41BA	I	−	+	−	−
PM0741	89.4	TonB dependent outer membrane haemoglobin/haemin binding receptor	pDEST-17	I	+	+	−	−
PM0778	42.9	HexD - putative polysaccharide export protein wza precursor	pDEST-41BA	S	+	+	+	−
PM0786	37.9	OmpA - β-barrel membrane anchor protein	pDEST-17	I	+	+	+	−
PM0803	90.3	Pm0803 - iron regulated outer membrane protein	pDEST-17	I	+	+	−	−
PM0881	18.4	Pm0881 - hypothetical outer membrane protein	pDEST-17	I	+	+	−	−
PM0892	17.1	ImpA - hypothetical outer membrane protein	pDEST-41BA	I	−	+	−	−
PM0903	45.5	Pm0903 - hypothetical outer membrane protein	pDEST-17	I	+	+	−	−
PM0928	41.2	MltA - murein transglycosylase A	pDEST-17	I	+	+	−	−
PM0966	16.1	Omp16 - Outer membrane protein P6 precursor; peptidoglycan-associated outer membrane lipoprotein	pDEST-17	I	+	+	+	−
PM0979	14.5	Pm0979 - immunogenic lipoprotein	pDEST-17	I	+	+	−	−
PM0998	30.6	Pm0998 - uncharacterised outer membrane protein	pDEST-41BA	I	+	+	+	−
PM1016	42.1	Wza - putative polysaccharide export protein	pDEST-17	I	+	+	+	−
PM1025	20.4	Opa - outer membrane protein	pDEST-17	I	+	+	+	−
PM1050	37.2	Pm1050 - uncharacterised lipoprotein	pDEST-41BA	S	+	+	+	−
PM1064	30	Pm1064 - uncharacterised lipoprotein	pBAD-DEST49	I	+	+	−	−
PM1069	47.7	Pm1069 - outer membrane protein P1 precursor	pDEST-41BA	I	+	+	+	−
PM1077	20.4	Pm1077 - hypothetical protein	pDEST-41BA	S	+	+	+	−
PM1081	90.8	Pm1081 - TonB dependent outer membrane haemoglobin/haemin binding receptor	pDEST-41BA	I	+	+	+	−
PM1225	49.1	ComE - putative competence protein E	pDEST-17	I	+	+	−	−
PM1238	36.9	Pm1238 - putative O-sialoglycoprotein endopeptidase	pDEST-17	I	−	+	+	−
PM1245	32.7	Pm1245 - putative L-xylulose 5-phosphate 3-epimerase	pDEST-41BA	I	−	+	−	−
PM1426	35.4	Phospholipase A - OMPLA	pDEST-41BA	I	+	+	+	−
PM1428	90.7	Pm1428 - TonB dependent outer membrane haemoglobin/haemin binding receptor	pBAD-DEST49	I	+	+	+	−
PM1451	30.4	XynC - predicted esterase	pDEST-41BA	I	+	+	+	−
PM1501	27.4	VacJ lipoprotein homolog, Surface lipoprotein *H. influenzae*	pDEST-17	I	+	+	−	−
**PM1517**	**37.3**	**PlpE - protective outer membrane lipoprotein**	**pDEST-17**	**I**	**+**	**+**	**+**	**+**
PM1578	35.7	Pm1578 - uncharacterised lipoprotein	pDEST-41BA	S	+	+	+	−
PM1611	19.6	PM1611 - hypothetical protein	pDEST-17	S	+	+	−	−
PM1614	49.6	Pm1614 - outer membrane antigenic lipoprotein B	pDEST-17	S	+	+	−	−
PM1622	95.8	HasR - Ton B dependent haeme acquisition system receptor	pDEST-17	I	+	+	+	−
PM1670	22.9	RpiA - ribose-5-phosphate isomerase A	pDEST-41BA	S	−	+	−	−
PM1707	40.1	PM1707 - uncharacterised protein	pDEST-41BA	S	+	+	+	−
PM1717	95.1	PM1717 - uncharacterised protein	pBAD-DEST49	I	+	+	−	−
PM1720	29.2	Pm1720 - DNA uptake lipoprotein	pDEST-17	I	+	+	+	−
PM1730	30.1	MetQ - periplasmic methionine binding protein of ABC transporter	pDEST-41BA	I	+	+	+	−
PM1809	67	Pm1809 - uncharacterised outer membrane protein	pDEST-17	I	+	+	+	−
PM1826	25.5	Pm1826 - uncharacterised lipoprotein	pDEST-41BA	I	+	+	+	−
PM1886	15.4	Pm1886 - uncharacterised outer membrane protein	pDEST-17	I	+	+	−	−
PM1898	22.4	Pm1898 - uncharacterised lipoprotein	pDEST-41BA	S	−	+	−	−
PM1974	15.7	Pm1974 - LysM domain/BON superfamily protein	pDEST-17	I	−	+	+	−
PM1980	51.8	TolC2 - outer membrane efflux channel	pDEST-41BA	I	+	+	−	−
PM1992	87.6	Oma87– Omp85-like protein assembly protein	pDEST-17	I	+	+	+	−
PM1993	21.3	Skp - Skp export factor homolog	pDEST-17	I	+	+	+	−
PM1979	69.3	Pm1979 - peptidyl-prolyl cis-trans isomerase	pDEST-41BA	S	+	+	−	−
PM2008	20.8	Pm2008 - putative fimbrial biogenesis and twitching motility protein	pDEST-17	I	+	+	+	−

a - Gene numbers are from the Pm70 strain [28] homolog of the X-73 genes amplified and cloned.

b - Expression vectors: pBAD-DEST49, a Gateway® destination vector that features a thioredoxin fusion tag; pDEST-41BA a modified Gateway-adapted expression vector containing a NusA solubility tag [20] (originally derived from pLIC-NusA [48]); pDEST-17, a Gateway® destination vector for production of tagless native protein.

c - I  =  insoluble; S  =  soluble protein.

d - SignalP server signal peptide prediction. In general PCR primers were designed so as to amplify the gene region encoding the mature length protein, excluding the signal sequence, except where a signal sequence could not be predicted; in this case the primers were designed to encompass the entire gene.

e - immunogenic reaction against the recombinant protein.

f - serum reacted with a protein of appropriate size in a whole cell lysate derived from *P. multocida* grown *in vitro* at 37°C.

g - positive (X-73 whole killed cells) and negative controls used in the vaccine trial.

h - Due to its large size, PM0714 was purified in three overlapping sub-fragments spanning the entire protein.

### Vaccination and Challenge Studies in Chickens

Seventy-one recombinant proteins (as detailed in Table 1) were tested for their ability to protect against fowl cholera. Groups of chickens, 12–14 weeks of age, were vaccinated with two doses of 100 µg of each of the recombinant purified proteins and subsequently challenged with 10^3^ CFU *P. multocida* X-73 (∼10×ID_50_) in the first and second experiments. In the third experiment 300 CFU *P. multocida* VP161 (∼30×ID_50_) was the challenge dose. A killed whole cell bacterin (two doses of killed *P. multocida* X-73 or VP161) was used as a positive control. As a negative control, groups of chickens were immunised with adjuvant only. All of the 71 recombinant antigens elicited a strong immune response in chickens but only PlpE (which was purified in the denatured form in 8 M urea) stimulated protection against fowl cholera. Of the vaccinated chickens, 100% (7/7) survived the homologous *P. multocida* X-73 challenge (Table 2) and showed no clinical signs of infection. A second highly virulent *P. multocida* strain VP161 was also tested for protective efficacy and showed significant protection (5/10) (Table 2). All other vaccinated birds developed acute fowl cholera and were euthanized. Antibody responses against each protein were determined by western blot; all immunised chickens produced antibodies that reacted against the corresponding recombinant protein. There was no apparent difference in the responses to individual proteins (data not shown). The bacterin vaccinated group also reacted strongly against a range of different antigens. When the sera were tested by western blot for reaction against *P. multocida* X-73 whole cell lysate, only 32 of the 71 sera reacted with a band corresponding to the size expected for the native protein (Table 1).

**Table 2:** Protective capacity of 8 M urea-denatured, recombinant PlpE in chickens and mice.

Species	Experiment^a^	Immunogen	Challenge		Survival^b^	P value^c^
			Strain	Dose (CFU)	No. surviving/total	
chicken	2	adjuvant control	X73	10^3^	0/7	
		killed X-73 control		10^3^	7/7	<0.001
		PlpE		10^3^	7/7	<0.001
chicken	3	adjuvant control	VP161	300	0/10	
		killed Vp161 control		300	7/10	<0.001
		killed X73 control		300	3/10	
		PlpE		300	5/10	0.03
mouse	1	adjuvant control	X73	34	0/10	
		killed X73 control		60	5/5	<0.001
		PlpE		34	7/10	0.003
mouse	2	adjuvant control	X73	44	0/10	
		PlpE		44	7/9	<0.001

a Results of chicken experiment 1 are shown in Table 1.

b All animals deemed incapable of survival were euthanized in accordance with animal ethics requirements.

c Compared to adjuvant control: Fisher’s exact test.

### Vaccination and Challenge Studies in Mice

The recombinant PlpE protein was also tested for protective efficacy in mice. In the groups immunised with PlpE, 70 and 78% of the mice survived until the end of the trial following challenge with *P. multocida* X-73 (Table 2). However, there were no survivors in the control adjuvant groups.

### Characterization of a *plpE* Mutant in *P. multocida*

**Figure 1:**
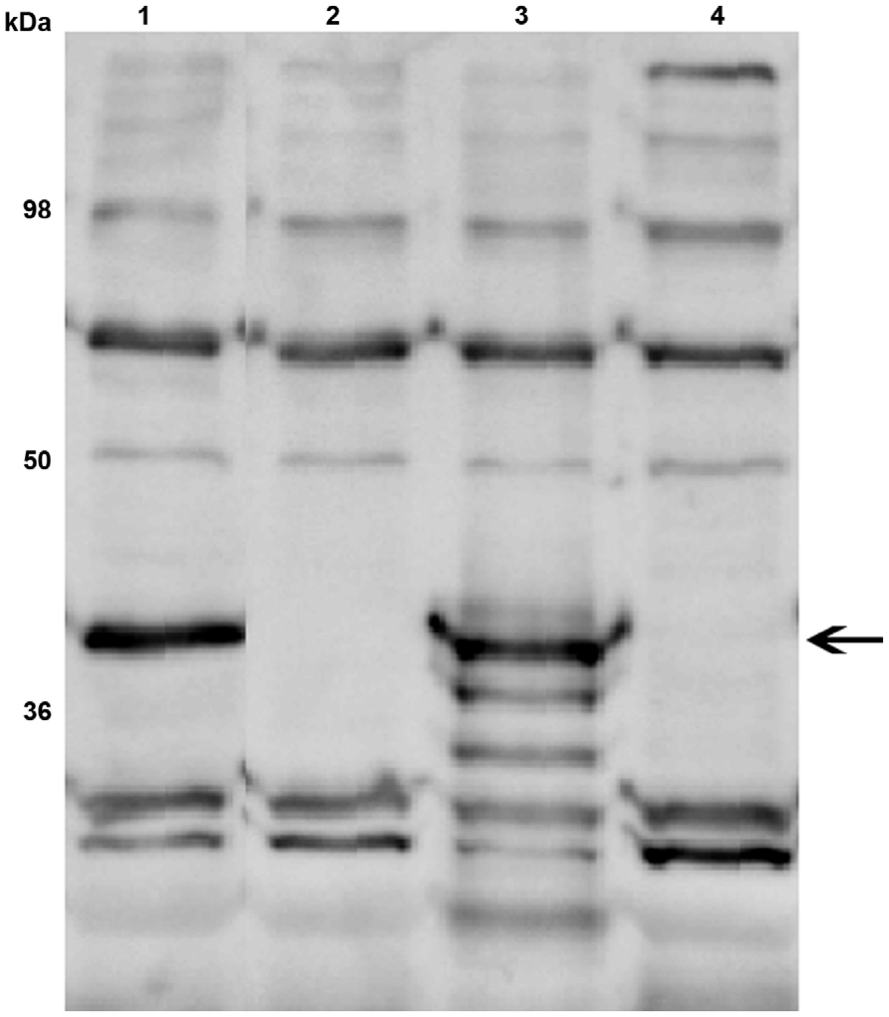
Western immunoblot analysis of PlpE expression in *P. multocida* whole cell lysates probed with chicken antiserum against recombinant PlpE. Lanes: wild type strain (lane 1); *plpE* mutant (lane 2); complemented mutant (lane 3); mutant transformed with empty vector (lane 4). Numbers on the left indicate the positions of molecular mass standards (in kDa). Arrow indicates the position of the 39 kDa PlpE. Pre-bleed serum showed no reactivity (data not shown).

In order to analyse the importance of PlpE for *P. multocida* virulence we constructed a *plpE* mutant by single-crossover insertional mutagenesis in the highly virulent *P. multocida* strain VP161. We were unable to generate a *plpE* mutant in the strain X-73, which, in our hands, was very refractive to genetic manipulation. The VP161 *plpE* mutant was complemented with an intact copy of the *plpE* gene cloned into the *P. multocida* expression plasmid. Western blot analysis was performed to confirm the protein profile of the *plpE* mutant and the complemented strain. Chicken antiserum raised against recombinant PlpE detected a band of 39 kDa in the wild-type VP161, consistent with the calculated molecular mass of mature PlpE (Fig. 1). The band was absent in the mutant and was restored by introduction of an intact *plpE* gene in the complemented strain (Fig. 1), but not in the mutant strain transformed with vector only. These data confirmed that the *plpE* mutant expressed no PlpE protein.

### Immunofluorescence Microscopy of PlpE

**Figure 2:**
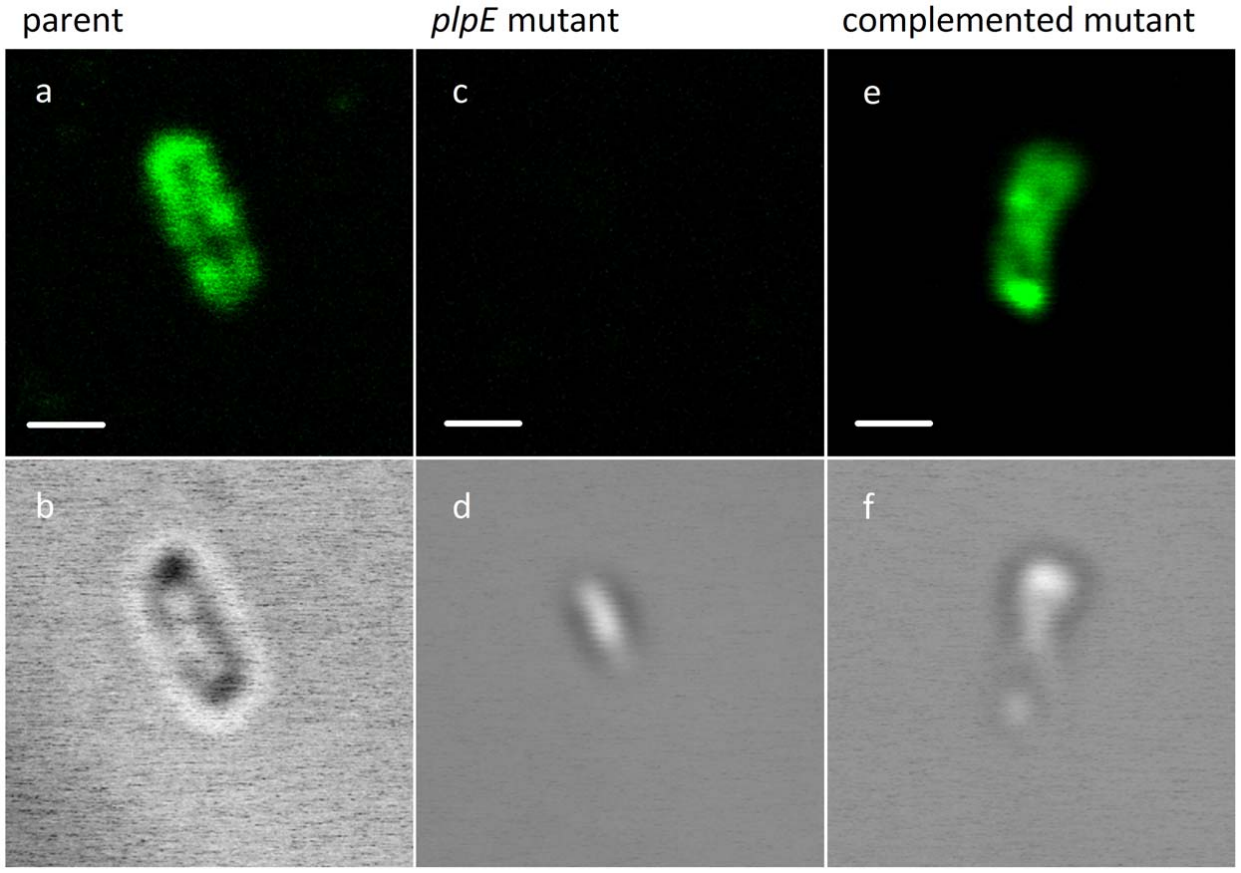
Surface localisation of *P. multocida* PlpE by immunofluorescence assay. Bacteria were fixed with paraformaldehyde, incubated with chicken antiserum against PlpE, stained with Alexa Fluor 488 goat anti-chicken IgG, and visualized by fluorescence microscopy. Fluorescence (panels: a, c, e) and DIC (Differential Interference Contrast) (panels: b, d, f) selected images of the bacterial Z-stack cross-sections. Control staining with antiserum raised against an unrelated protein showed no surface fluorescence (data not shown). Scale bar  = 1 µm.

The localisation of the PlpE protein to the surface of the wild-type *P. multocida* and complemented *plpE* mutant cells was confirmed by immunofluorescense labelling of *P. multocida* and visualisation by confocal laser scanning microscopy (Fig. 2). The Z-stack cross-sections of the bacteria (Fig. 2a, 2c) showed stained PlpE as dots scattered only on the bacterial surface and not in the cytosol. The *plpE* mutant showed no fluorescence, neither at the cell surface nor throughout the cytosol.

### Virulence of the plpE Mutant

Groups of four Balb/C female mice were infected with the *P. multocida* parent or *plpE* mutant. The disease progression for wild type and *plpE* mutant infected groups was identical, with all mice succumbing to infection, necessitating euthanasia after 16–19 hours (p = 1.0). Bacteria recovered from the blood of the infected mice retained the *plpE* mutation.

## Discussion

Recently, sequencing of whole bacterial genomes has allowed the development of new approaches to vaccine development. The bioinformatics analysis of genomic data can now readily be used to predict surface proteins, which can then be screened to identify new vaccine antigens [22]. This genome-based approach to vaccine development, coined “reverse vaccinology” [23], places an emphasis on identifying and validating the immunoreactivity and protective efficacy of bacterial antigens for use as recombinant vaccines against pathogens of animals and humans using rational deductive methodologies.

Subunit vaccines can be based on recombinant proteins, peptides or polysaccharides that have been shown to contain protective epitopes [1]. An example of a commercially available recombinant subunit vaccine is the Hib vaccine against *Haemophilus influenzae* type b. It contains a polysaccharide antigen conjugated to a carrier protein such as tetanus toxoid [24]. Conjugation to protein carriers enables induction of a T cell mediated immune response, even in children under two years of age [25], [26]. A second generation acellular *Actinobacillus pleuropneumoniae* subunit vaccine has been developed to include the five recombinant proteins, ApxII, TbpB, CsyL, OmlA(1) and OmlA(2) (PleuroStar APP). The vaccine confers some degree of cross-protection against all *A. pleuropneumonia* serotypes in pigs [18], [27].

We have used a range of bioinformatics analyses of the annotated *P. multocida* Pm70 genome [28] sequence and previously published experimental data to select genes encoding proteins likely to have vaccine potential [20]. Protective antigens are likely to be surface exposed or secreted by the bacteria and therefore accessible to the host immune system. On this basis we chose 71 proteins that were identified as predicted OM-associated or secreted proteins [20] to test for their immunogenicity and protective efficacy. This list contained 13 lipoproteins, 11 previously characterised proteins with functions such as porins, membrane transporters or secretory proteins, seven haem or haemoglobin receptor-related proteins, several proteins with putative functions and 17 uncharacterized proteins. Given the average identification rate of protective antigens from previous reverse vaccinology studies [2]–[5], we predicted that 1–2 protective antigens might be discovered.

All of the 71 recombinant proteins produced a strong immune response in chickens as all tested sera recognised the appropriate recombinant proteins on a western immunoblot. However, more than half of the sera failed to react conclusively with the protein in the *P. multocida* whole cell lysates, suggesting that many proteins are expressed exclusively *in vivo* or that the antigen could not be detected with *in vitro* grown bacteria. Importantly, out of the 71 proteins tested only the denatured, urea-solubilised, recombinant PlpE could protect chickens and mice from infection with *P. multocida*. Previously, soluble PlpE was identified [29] as a protective antigen against *P. multocida* infection of chickens and mice against lethal challenge. PlpE shares 22% amino acid identity with a lipoprotein designated *Pasteurella* lipoprotein E (PlpE), from *Mannheimia haemolytica* that was found to be highly immunogenic in cattle [30]. PlpE is a lipid-modified, surface-exposed outer membrane protein that is important in complement mediated killing of *M. haemolytica* [31]. Addition of recombinant PlpE to the commercial *M. haemolytica* vaccine markedly enhanced the vaccine induced-resistance in calves against experimental challenge with serotypes 1 and 6 [30], [32]. We were able to purify PlpE only in denatured urea-solubilised form and this form conferred excellent levels of protection. Therefore we hypothesize that linear epitopes are sufficient to elicit the protective immune response. These linear epitopes may confer cross-strain protective immunity, since PlpE is 90–100% identical across all reported *P. multocida* strains [29], [33].

To analyse the localisation and importance in virulence we constructed a *P. multocida plpE* mutant and tested it in direct virulence assays. Blood was recovered from each infected mouse and tested for stability of the mutation. All of the recovered colonies contained the mutation. Therefore PlpE is stable under the conditions tested and therefore not essential for virulence. Using immunofluorescence analysis of the wild type strain and the *plpE* mutant strain we showed experimentally that PlpE is localized on the surface (Fig. 2), a location consistent with its protective properties.

Many of the protective antigens defined by reverse vaccinology are proteins that mediate virulence functions such as motility and attachment (pili proteins from group *B. streptococcus* [34] and *Neisseria* adhesin A, (NadA) [35]), complement evasion (*Neisseria* factor H binding protein [36]) and autolytic activity (LytB and LytC from *Streptococcus pneumoniae* [37], [38]). In *P. multocida* the major outer membrane porin OmpH and the filamentous haemagglutinin protein PfhB2 have been identified as partially protective and having a role in virulence [39]–[42]. However, the *plpE* mutant retained virulence in a mouse infection model. Therefore, we hypothesize that PlpE is involved in infection, but is not essential. Recent work in determining the function of *N. meningiditis* genes identified as protective antigens has shown that only one of the five (*Neisseria* factor H binding protein) is an essential virulence factor. However, a *nadA* mutant showed a decreased ability to bind to human epithelial cells *in vitro* [35] and another of the mutants showed reduced fitness *in vitro* [43]. Likewise, future studies may define a role for the protective antigen, PlpE, in the pathogenesis of pasteurellosis.

Despite the fact that the biological function of PlpE remains elusive, it is clear that the protein can stimulate protection against experimental challenge with *P. multocida* in both soluble and denatured urea-solubilised forms. Furthermore, this antigen can give 100% protection against the homologous strain and significant 50% protection against a second highly virulent strain. Further studies are required to demonstrate the level of cross-protection provided by denatured PlpE against a wide range of *P. multocida* serovars. Further, this is the first documented report to our knowledge where a denatured recombinant protein has elicited high levels of protection against fowl cholera.

## Materials and Methods

### Ethics Statement

Animal experimentation was approved by the Monash University School of Biomedical Sciences Animal Ethics Committee.

### Bacterial Strains, Plasmids, Media and Growth Conditions

The bacterial strains and plasmids used in this study are listed in Table 3. *Escherichia coli* was grown in Luria-Bertani broth at 37°C. *P. multocida* strains were grown in either brain heart infusion (BHI) broth or nutrient broth (NB) (Oxoid, Basingstoke, U.K) supplemented with yeast extract (3% w/v) at 37°C with agitation. Solid media were obtained by the addition of 1.5% (w/v) agar. When required, media were supplemented with spectinomycin (50 µg/ml), streptomycin (50 µg/ml), kanamycin (50 µg/ml) or tetracycline (2.5 µg/ml for routine culturing or 8 µg/ml for selection of *P. multocida* transconjugants).

**Table 3:** Bacterial strains and plasmids used in this study.

Strain or plasmid	Relevant description	Source or reference
**Strains**		
***P. multocida***		
X-73	Serotype A:1, wild type strain	[54]
VP161	Serotype A:1, Vietnamese chicken isolate	[55]
AL1119	VP161 carrying a Tn*916* insertion in gene *pm1417.* Fully virulent	This study
AL1172	*Pm1517 (plpE)* mutant of AL1119	This study
AL1174	AL1172 containing pAL617	This study
AL1175	AL1172 containing pAL99	This study
***E. coli***		
DH5α	*deoR*, *endA*1, *gyrA*96, *hsdR*17(r_k_^-^ m_k_^+^), *recA*1, *relA*1, *supE*44, *thi*-1, (*lacZYA*-*argF*V169), Φ80*lacZ* ΔM15, F-	Bethesda Research Laboratories
Sm10 l *pir*	Strain for propagation of pUA826 and its derivatives	[56]
Expression strains	For all recombinant expression strains see Table 1	This study
		
**Plasmids**		
pUA826	Mob+, R6K replicon, ApR StrR SpcR. Single-crossover insertional mutagenesis vector	[57]
pAL99	*P. multocida* expression plasmid (KanR)Constitutive tpiA promoter for expression of cloned genes	[58]
pAL543	Internal section of *pm1517 (plpE)* gene cloned into pUA826	This study
pAL617	Complete *pm1517 (plpE)* gene cloned into pAL99	This study

### Selection of Vaccine Target Antigens

PSORTB [44] and ProteomeAnalyst [45] were used to predict outer membrane and secreted proteins. LipoP [46] was used to predict lipoproteins. All lipoproteins were included since many lipoproteins are observed in the *P. multocida* outer membrane proteome [47] and sub-cellular prediction algorithms for lipoproteins may be unreliable. Additionally the scientific literature was mined to find additional proteins with vaccine potential [20].

### Cloning of Candidate Antigen Genes

The large number of *P. multocida* proteins that were identified as selected vaccine target antigens necessitated the adoption of a high-throughput cloning strategy using the Gateway (Invitrogen Inc., Carlsbad, CA, USA) cloning and expression system to clone PCR-amplified *P. multocida* ORFs. In general, PCR primers were designed to amplify the gene region encoding the mature length protein, excluding the signal sequence, except where a signal sequence could not be predicted; in this case the primers were designed to encompass the entire gene. All 5' PCR primers included a 5'-CACC tail to facilitate directional topoisomerase cloning and where the primers had been designed to amplify only the mature length portion (without the signal sequence) the 5'-CACCATG tail was added so as to include a start codon. Due to its size, the protein Pm0714 was purified in three overlapping subfragments spanning the entire protein. The primers used are listed in supplementary Table S1. As the genes were to be expressed in-frame with either a C-terminal or an N-terminal tag, the native stop codon was not included in the reverse primers. Genes were cloned into the Gateway entry vector pENTR/SD/D topo and all cloned fragments confirmed by DNA sequencing. The cloned genes were then transferred to the expression vectors pBAD-DEST49™, pDEST-17™ and pDEST-41BA (a modified Gateway-adapted expression vector containing a NusA solubility tag [20], originally derived from pLIC-NusA [48]) (Table 1). The particular expression construct used for each gene is shown in Table 1. Expression vectors were transformed into the expression host strain *E. coli* BL21 codon plus (Stratagene, Agilent Technologies, Inc., CA, USA).

### Expression and Purification of Antigens

For the expression of recombinant proteins, 200 ml cultures (Overnight Express™, Merck, NJ, USA) containing ampicillin (100 µg/ml) were grown overnight at 28°C, with constant shaking at 250 rpm. The cells were collected by centrifugation at 3500 *g* for 10 min and resuspended in nickel affinity buffer (100 mM sodium phosphate buffer, pH 7.4, containing 150 mM NaCl and 10 mM imidazole). The cells were then lysed by sonication on ice for six rounds of 30 sec with a 10 mm sonication probe, interspersed with 30 sec rest intervals. After sonication the soluble and insoluble fractions were separated by centrifugation at 7500 *g* for 20 min.

For the soluble proteins, the soluble fraction prepared above was filtered through a 0.22 µm filter and loaded on to a HisTrap FF nickel affinity column (GE Healthcare – Life Sciences) at a flow rate of 1 ml/min. After washing the column with the nickel affinity buffer, the recombinant proteins were eluted from the nickel affinity column with 100 mM sodium phosphate buffer, pH 7.4, containing 150 mM NaCl and 0.5 M imidazole. The eluted proteins were then loaded on to a HiLoad 16/60 Superdex 200 size exclusion chromatography column (GE Healthcare – Life Sciences) and the fractions containing the protein of interest were collected in 100 mM sodium phosphate buffer, pH 7.4, containing 150 mM NaCl.

For the insoluble proteins, the insoluble fraction was washed twice with 100 mM sodium phosphate buffer, pH 7.4, with 150 mM NaCl and 1 mM 2-mercaptoethanol, 1% (v/v) Triton X-100 (centrifugation at 7500* g* for 20 min). The third wash was with 100 mM sodium phosphate buffer, pH 7.4, with 150 mM NaCl and 1 mM 2-mercaptoethanol and the insoluble fraction again pelleted by centrifugation at 7500 *g* for 20 min. The pellet was resuspended in 100 mM sodium phosphate buffer, pH 7.4, with 150 mM NaCl, 10 mM 2-mercaptoethanol and 8 M urea and mixed for one hour at room temperature. Any proteins that remained insoluble after this incubation period were removed by centrifugation at 27000 *g* for 20 min, and the urea-solubilized material was loaded on to a HisTrap FF column at 1 ml/min. After washing this column with 100 mM sodium phosphate buffer, pH 7.4, with 150 mM NaCl, 1 mM 2-mercaptoethanol and 8 M urea, the recombinant proteins were eluted with the same buffer containing 0.5 M imidazole. The eluted proteins were loaded on to a Hiload 16/60 desalting column (GE Healthcare - Life Sciences) and the various fractions collected in 100 mM sodium phosphate buffer, pH 7.4, with 150 mM NaCl, 1 mM 2-mercaptoethanol, and 8 M urea.

The various fractions from both methods were analysed by visualization of the recombinant proteins using SDS-PAGE gels stained with Coomassie blue. The concentration was quantified using Bradford assay (Biorad, CA, USA).

### Vaccination and Challenge Studies in Chickens

A total of 71 candidate antigens were tested in 12-week-old commercial Hy-line layer hens. In an initial experiment birds were randomly assigned to 24 pens with 12 birds in each pen. All birds were wing-tagged. Each treatment group consisted of four birds, so each pen housed three treatment groups. In a second experiment, single treatment groups of seven birds in separate pens were immunised with recombinant PM0527, PM0680, PM1426, PM1707 and denatured urea-solubilised recombinant PM1517 (PlpE). In a third experiment, groups of 10 birds in separate pens were immunised with recombinant, denatured, urea-solubilised recombinant PM1517 (PlpE).

Chickens were vaccinated subcutaneously with 0.5 ml of vaccine containing 100 µg purified recombinant protein with 20% Alhydrogel v/v (Sigma). The vaccination was repeated two weeks later. For each experiment, a group of birds acted as a negative control and were mock vaccinated with adjuvant. At 16 weeks of age all birds were challenged by intramuscular injection with 100 µl of bacterial culture containing, in the first and second experiments, 10^3^ CFU of *P. multocida* strain X-73 in BHI (ID_50_ = 100 CFU). In the third experiment 300 CFU of *P. multocida* strain VP161 in BHI (ID_50_ =  <10 CFU) was used for challenge. All birds that developed signs of acute fowl cholera were euthanized in accordance with animal ethics requirements. Pre-challenge serum was collected from all birds.

### Vaccination and Challenge Studies in Mice

The protective efficacy of the denatured PlpE protein was also tested in mice. Groups of 10 female 6–8-week-old Balb/C mice were immunised subcutaneously twice with 150 µg of urea-solubilised recombinant PlpE mixed with Alhydrogel 20% v/v and challenged by intraperitoneal injection with approximately 40 CFU (100 µl) of virulent *P. multocida* X-73. Sera were collected before the challenge. All animals that developed signs of acute infection were euthanized in accordance with animal ethics requirements.

### DNA Manipulations

Restriction digests and ligations were performed according to the manufacturers’ instructions using enzymes obtained from NEB (Beverley, MA) or Roche Diagnostics GmbH (Mannheim, Germany). Plasmid DNA was prepared using alkaline lysis [49] and further purified using Qiagen columns (QIAGEN GmbH, Germany), while genomic DNA was prepared using the CTAB method [50]. PCR amplification (95°C - 5 min; 30 cycles of 95°C - 30 s, 54°C - 30 s, 72°C - 2 min; finally 72°C - 5 min) of DNA was performed using *Taq* DNA polymerase (Roche Diagnostics GmbH) or the Expand High Fidelity PCR System (Roche Diagnostics GmbH) and purified using the Qiagen PCR Purification Kit. The oligonucleotides used in this study are listed in supplementary Table S1. DNA sequences were determined on an Applied Biosystems 3730S Genetic Analyser and analysed with Sequencher Version 3.1.1 (GenCodes, Ann Arbor, MI, USA).

### Construction of a *P. multocida plpE* Mutant

A *plpE (pm1517)* mutant was constructed in a tetracycline resistant derivative of *P. multocida* VP161 (strain AL1119; Table 3) as described previously [51] using a single-crossover insertional mutagenesis procedure. Briefly, an internal DNA fragment of *plpE* was amplified by PCR, using the primers listed in supplementary Table S1, digested with *Sal*I, and ligated into *Sal*I-digested pUA826 (Table 3). To generate the donor strain, the ligation mix was transformed into *E. coli* SM10 λ *pir* cells and then transformants were screened by PCR for recombinant pUA826 with internal and flanking primers. The correct recombinant plasmid (Table 3) was then mobilized into the recipient *P. multocida* strain AL1119 by conjugation [51]. Single crossover insertion of the recombinant plasmid into the target genes was confirmed by PCR using flanking primers in combination with primers located within the vector sequence (supplementary Table S1). Mutants constructed previously in identical fashion were shown to be stable [51], [52].

### *In trans* Complementation of Mutants

For complementation of the *plpE* mutant an intact copy of *plpE* was amplified from *P. multocida* VP161 genomic DNA using flanking oligonucleotides (supplementary Table S1). The amplified DNA fragments were ligated into *Sal*I- and *Bam*HI-digested expression vector pAL99 (Table 3), such that transcription of the gene would be driven by the constitutive *P. multocida tpiA* promoter. The nucleotide sequence of the recombinant plasmid was determined to check fidelity of the cloned gene, and subsequently introduced by transformation into the *P. multocida plpE* mutant, generating the complemented strain AL1174 (Table 3). As a control, the vector pAL99 was introduced separately into each mutant (Table 3).

### Immunoblotting

To prepare whole-cell lysates, *P. multocida* strains (Table 3) were grown to late logarithmic phase (3×10^9^ CFU/ml) in BHI broth containing the required antibiotics, harvested by centrifugation, and lysed by boiling for 5 min in SDS-PAGE sample buffer. Approximately 10^8^ CFU equivalents were loaded in each lane and separated by SDS-PAGE, transferred to nitrocellulose membranes, incubated with chicken antiserum raised against the recombinant PlpE (1∶2500) and detected using peroxidase-conjugated anti-chicken antibody (1∶2500) (Chemicon International Inc., Temecula, CA, USA). Reactions were detected using ECL (GE Healthcare, UK).

### Localization of *PlpE* by Confocal Microscopy

For immunofluorescence analysis of the localization of *P. multocida* PlpE, wild type, *plpE* mutant, or complemented mutant strains (Table 3) were grown to mid logarithmic phase (OD_600_ = 0.2), pelleted by centrifugation, washed with PBS and then fixed with paraformaldehyde [53]. After blocking for 30 min in PBS containing 1% bovine serum albumin (BSA), bacteria were incubated with a 1∶2500 dilution of anti-PlpE chicken serum in PBS with 1% BSA for 1 h at 37°C. After washing three times in PBS, the bacteria were labelled with Alexa Fluor 488 goat anti-chicken IgG (Invitrogen) at a dilution of 1∶250 in PBS with 1% BSA for 1 h at 37°C. Stained cells were washed with PBS, coverslips mounted with aqueous mounting medium and visualised using a confocal laser scanning microscope (CLSM; Olympus FV500) equipped with a 1.2 NA water immersion lens (Olympus 60× UPlanapo). Image analysis and processing were performed using Olympus FluoView TIEMPO software (Version 4.3) and the public domain software Image-J 1.41a. The surface localization of PlpE was assessed using Z-stack CLSM analysis.

### Virulence Trials with *plpE* Mutant

Groups of four Balb/C female mice, 6–8 weeks old, were infected with approximately 100 CFU of *P. multocida* parent or *plpE* mutant strains by injection of 100 µl intraperitoneally. Blood samples were obtained at the end point of the trial or when the mice were deemed incapable of survival, at which time they were euthanized in accordance with animal ethics requirements. Blood samples were diluted two-fold in BHI broth containing heparin and plated onto BHI plates. *P. multocida* colonies isolated from the blood were patched onto NB agar and NB agar with the appropriate antibiotics. Colonies recovered from mice injected with the *plpE* mutant strain grew on plates containing tetracycline, streptomycin and spectinomycin, a phenotype consistent with retention of the *plpE* mutation.

## Supporting Information

Table S1 **Oligonucleotides used in this study.**
(DOC)
