# Supplementary material for: Screening of 71 P. multocida Proteins for Protective Efficacy in a Fowl Cholera Infection Model and Characterization of the Protective Antigen PlpE
Source: PLoS One. 2012 Jul 5;7(7):e39973. doi: 10.1371/journal.pone.0039973 (PMC3390355; doi:10.1371/journal.pone.0039973)
Supplement: Table S1 — Oligonucleotides used in this study. (DOC) [file pone.0039973.s001.doc]

**Supplementary Table S1**

Oligonucleotides used in this study.

| **Oligo** | **Sequence (5’-3’)** | **Description** |
| --- | --- | --- |
| PM1517(150) | ACGCGTCGACCGCTCCAATCAAAAATCCTAC | *pm1517 (plpE*) internal forward primer for mutagenesis |
| PM1517(901) | ACGCGTCGACTCAGCTCCATTGCCATGTTGTC | *pm1517 (plpE)* internal reverse primer for mutagenesis |
| BAP5248 | GGGCCCGGATCCATGCCGCAGTTCAGCTTATT | *pm1517 (plpE)* flanking forward primer for expression (complementation) |
| BAP5249 | GGGCCCGTCGACGCGGTATGCGAAAATACCAT | *pm1517 (plpE)* flanking reverse primer for expression (complementation) |
| BAP2610 | CACCATGGAAAACGCGGCTACCACG | PM0040 - forward primer for protein expression |
| BAP2611 | AAAGTGGTAACGAATATTGGC | PM0040 - reverse primer for protein expression |
| BAP2610 | CACCATGGAAAACGCGGCTACCACG | PM0040 - forward primer for protein expression |
| BAP2611 | AAAGTGGTAACGAATATTGGC | PM0040 - reverse primer for protein expression |
| BAP4250 | CACCATGGATTTACCTTTTCACGATGACGCG | PM0055 - forward primer for protein expression |
| BAP4251 | AACATCAAATGGAAAATGTGTTTTGATG | PM0055 - reverse primer for protein expression |
| BAP2922 | CACCATGTTAAATTTCGATGAAGTGAGTCAGC | PM0056 - forward primer for protein expression |
| BAP2921 | AAAAGAAACAGAACCACTGACG | PM0056 - reverse primer for protein expression |
| BAP3057 | CACCATGAAAGATGTTGTCGTGTTTGGTGATA | PM0076 - forward primer for protein expression |
| BAP3058 | GAAGGTAAAACCAATGCCCG | PM0076 - reverse primer for protein expression |
| BAP2914 | CACCATGGTGGATTCAGAAAAACACACTC | PM0098 - forward primer for protein expression |
| BAP2913 | TTTTTTATAAATATAACTGCCATCGGC | PM0098 - reverse primer for protein expression |
| BAP2912 | CACCATGAAAAAAGAAGAAGGGCAAGATTC | PM0243 - forward primer for protein expression |
| BAP2911 | TAGTCTGAGCTGCCGCTCAATTTC | PM0243 - reverse primer for protein expression |
| BAP2602 | CACCATGACCCTGGATACAGAACGTCCAAC | PM0246 - forward primer for protein expression |
| BAP2603 | ATAAGTCCAGTTATCGATACGAATC | PM0246 - reverse primer for protein expression |
| BAP2910 | CACCATGGATAGTCATCAAGAGGCGACTGA | PM0300 - forward primer for protein expression |
| BAP2909 | GAAGGTGATTTCGGCACTGA | PM0300 - reverse primer for protein expression |
| BAP2580 | CACCATGCATCAAGCGGGTAGTGTGA | PM0331 - forward primer for protein expression |
| BAP2581 | AAAACGATAACCTAAGCCAACAAAG | PM0331 - reverse primer for protein expression |
| BAP2906 | CACCATGGAAGAGGTAAAATCCACAGAGGC | PM0337 - forward primer for protein expression |
| BAP2905 | GAATGTGAATTCAGCATTTAAGCGGA | PM0337 - reverse primer for protein expression |
| BAP4264 | CACCATGTTTCACCACGAAGAGATGAATTATACG | PM0355 - forward primer for protein expression |
| BAP4265 | TTTGTTTGATATTAAAAATCTTTCAATTGC | PM0355 - reverse primer for protein expression |
| BAP2904 | CACCATGCAGTCTGCACCGGTTTCTCC | PM0368 - forward primer for protein expression |
| BAP2903 | TTGTAGGCGAGATAAACGCTC | PM0368 - reverse primer for protein expression |
| BAP2582 | CACCATGGCAACAGTTTACAATCAAGAC | PM0388 - forward primer for protein expression |
| BAP2583 | GAAGTGTACGCGTAAACCAACAC | PM0388 - reverse primer for protein expression |
| BAP2608 | CACCATGGCAACCGTTTTCGATAAAGAAGGA | PM0389 - forward primer for protein expression |
| BAP2609 | GAAGAACACGCGTAAACCTACAC | PM0389 - reverse primer for protein expression |
| BAP2586 | CACCATGTTTGATAAAGAGAGCAAAGCTG | PM0442 - forward primer for protein expression |
| BAP2587 | TTGTGCTTTAGGCACAATCAC | PM0442 - reverse primer for protein expression |
| BAP4270 | CACCATGTTGGCAAAAGATGGTCAATTTAT | PM0519 - forward primer for protein expression |
| BAP4271 | TGCTTCGTTTGTCAAATCTTTAATG | PM0519 - reverse primer for protein expression |
| BAP2588 | CACCATGGCTAATCTGGATGATTCTTATC | PM0527 - forward primer for protein expression |
| BAP2589 | TTTTGCTGAGTAATACCCTGCCA | PM0527 - reverse primer for protein expression |
| BAP4274 | CACCATGGTTGAGTTGTGTTTGTTCAGCGAA | PM0542 - forward primer for protein expression |
| BAP4275 | CTTATTTAACTGTTGCAACACACCAAAAG | PM0542 - reverse primer for protein expression |
| BAP2578 | CACCATGGCTAACACCGACATTTACAGTG | PM0554 - forward primer for protein expression |
| BAP2579 | ATGAATCACAGATACATTCACTTTAGAAC | PM0554 - reverse primer for protein expression |
| BAP2612 | CACCATGGGTAATTTAAGTAAAGTTACTCCA | PM0586 - forward primer for protein expression |
| BAP2613 | TTTTCTATAAAGTGGTGCTGGTCCTG | PM0586 - reverse primer for protein expression |
| BAP3059 | CACCATGAGTAAAGAATTCGA | PM0612 - forward primer for protein expression |
| BAP3060 | TTTCTTAGAGATTAAAACGCCTA | PM0612 - reverse primer for protein expression |
| BAP4278 | CACCATGCTTGATTTTCAACTTGATCCGAAAAAAACG | PM0618 - forward primer for protein expression |
| BAP4279 | TTGTAAGGCTTTTTCGATTTTCTC | PM0618 - reverse primer for protein expression |
| BAP2890 | CACCATGTGGGATAAAAAGCAAGATGAGGC | PM0659 - forward primer for protein expression |
| BAP2889 | TTTGCGGACTTTAATTTGTCCTTCAC | PM0659 - reverse primer for protein expression |
| BAP4286 | CACCATGATTGAACAATTACTCAAACAAGAGCAATTCAC | PM0680 - forward primer for protein expression |
| BAP4287 | ATTCGCAATGCCTTTATGTCTTAAAAGG | PM0680 - reverse primer for protein expression |
| BAP2884 | CACCATGATTACTGCCCCGAAAGGGCTTGA | PM0708 - forward primer for protein expression |
| BAP2883 | TTGCAACACATAGCGTAAGCG | PM0708 - reverse primer for protein expression |
| PM714-1 Fw | CACCATGGTCTCAGAGCTTGCAACC | PM0714-1 forward primer for protein expression |
| PM714-1 Rv | CACTGCGTTATAACCAATAGCGATC | PM0714-1 reverse primer for protein expression |
| PM714-2 Fw | CACCATGTCTGATAAAAATCCT | PM0714-2 forward primer for protein expression |
| PM714-2 Rv | TAAGCCATCGTTAAGCGTAGC | PM0714-2 reverse primer for protein expression |
| PM714-3 Fw | CACCATGATCGCAGGTCCAAAT\ | PM0714-3 forward primer for protein expression |
| PM714-3 Rv | CCATTGATAACCGATACTACC | PM0714-3 reverse primer for protein expression |
| BAP2882 | CACCATGCAAGAAACGACTGAATTAGAACA | PM0741 - forward primer for protein expression |
| BAP2881 | GAAACGGATTTCAAGGGAAG | PM0741 - reverse primer for protein expression |
| BAP2598 | CACCATGCATAGCATGCCAACTTCG | PM0778 - forward primer for protein expression |
| BAP2599 | TAAATTTTTAATTACATTAATATTATGAAG | PM0778 - reverse primer for protein expression |
| BAP2531 | CACCATGGCACCACAACCTAACAC | PM0786 - forward primer for protein expression |
| BAP2532 | TTTGTTACCTTTAACAGCGATTTC | PM0786 - reverse primer for protein expression |
| BAP2521 | CACCATGAAAACCGATACGGACTCCGA | PM0803 - forward primer for protein expression |
| BAP2522 | GAATTCGTAACCAACTTCAAGCCA | PM0803 - reverse primer for protein expression |
| BAP2870 | CACCATGAGTCGTTCGATACCTTGTCAG | PM0881 - forward primer for protein expression |
| BAP2869 | TTTTCTCTTTTGTCTGCGCCATTGC | PM0881 - reverse primer for protein expression |
| BAP4298 | CACCATGATGAATAGCATAAACAACACTGTGGCTC | PM0892 - forward primer for protein expression |
| BAP4299 | TGCCGCAAATGCCATATTGCGTTTTG | PM0892 - reverse primer for protein expression |
| BAP2868 | CACCATGAATAATGTGTGGACCATTGCTATTG | PM0903 - forward primer for protein expression |
| BAP2867 | ATCTCTCAATTTAATTTTTTGTCCTGTTAAC | PM0903 - reverse primer for protein expression |
| BAP2866 | CACCATGAGCTCGCAGCCTAGAGGAAGTGC | PM0928 - forward primer for protein expression |
| BAP2865 | TTGTAATACCCACACACGTCCATAATG | PM0928 - reverse primer for protein expression |
| BAP2533 | CACCATGGGTTCATCTAAAAAAGATGAAAGC | PM0966 - forward primer for protein expression |
| BAP2534 | GTATGCTAACACAGCACGAC | PM0966 - reverse primer for protein expression |
| BAP2862 | CACCATGGCACAAAATGGTGCGGACA | PM0979 - forward primer for protein expression |
| BAP2861 | TTTTGCGTTAGTACAATTTTTAGCTAG | PM0979 - reverse primer for protein expression |
| BAP2858 | CACCATGTGTTTAATGGCTCTTCCAATATCCAG | PM0998 - forward primer for protein expression |
| BAP2857 | AAACATATAGTAGAAATTTAAGGCTGAAC | PM0998 - reverse primer for protein expression |
| BAP2854 | CACCATGTCCGTCATGCTACCCTCTACC | PM1016 - forward primer for protein expression |
| BAP2853 | CCAATTTCGGATTCTTAACATACTTTCA | PM1016 - reverse primer for protein expression |
| PM1025 Fw | CACCATGAATTTTTATGTTCAGGGGGA | PM1025- forward primer for protein expression |
| PM1025 Rv | GAAATCGTAACGTAAACCCACTTTTGC | PM1025- reverse primer for protein expression |
| BAP2850 | CACCATGTCAGTGAGCAATGAAAGTAAACAGC | PM1050 - forward primer for protein expression |
| BAP2849 | TTTCGCTAAGAGATTTTGTAATGC | PM1050 - reverse primer for protein expression |
| BAP2523 | CACCATGTCAAATCAACAAGCGGTTGAG | PM1064 - forward primer for protein expression |
| BAP2524 | TTTACCCTCCCAAGCATGAATTG | PM1064 - reverse primer for protein expression |
| BAP2525 | CACCATGGCCGCTTTCCAACTAGCAG | PM1069 - forward primer for protein expression |
| BAP2526 | GAAACGGTAATTCACGTTTAAACCATA | PM1069 - reverse primer for protein expression |
| BAP2844 | CACCATGGATAATGCCAGCAACACGCCA | PM1077 - forward primer for protein expression |
| BAP2843 | TTTTTGTTGGTATTGCTGTTTTAG | PM1077 - reverse primer for protein expression |
| BAP2842 | CACCATGCAAAGCCCACTAAAAAATACAAG | PM1081 - forward primer for protein expression |
| BAP2841 | GAATTTAAAATTCATTCCCACATTAAGA | PM1081 - reverse primer for protein expression |
| BAP2594 | CACCATGCAAGACGCAGAACATTTTTATTTA | PM1225 - forward primer for protein expression |
| BAP2595 | CCTCGATTTTGCAGATTTTTTAACTC | PM1225 - reverse primer for protein expression |
| BAP2834 | CACCATGATGCGCGTGTTAGGCATTGA | PM1238 - forward primer for protein expression |
| BAP2833 | ATCAATCGCAGAAAGCTCAGTC | PM1238 - reverse primer for protein expression |
| BAP4304 | CACCATGTGTGGCTTTGACTTTGTTGAAATTTCC | PM1245 - forward primer for protein expression |
| BAP4305 | ACA TTG GAA ACC ACC TTC TTT CAT TTT TTG | PM1245 - reverse primer for protein expression |
| BAP2826 | CACCATGAATGAAAGTGACGCGCGTCCCTA | PM1426 - forward primer for protein expression |
| BAP2825 | GAAAACATTGTTCAAGGAAATGCCGA | PM1426 - reverse primer for protein expression |
| BAP2824 | CACCATGAATACAGAAAAGAAGCAAATTG | PM1428 - forward primer for protein expression |
| BAP2823 | GAAATGGATGTTTAATCCTAAAGTCACA | PM1428 - reverse primer for protein expression |
| BAP2820 | CACCATGTTTAGTGAAAAAAATGTGGAAGTC | PM1451 - forward primer for protein expression |
| BAP2819 | TTTCTCTTTAGCTTGTGCAAATTTC | PM1451 - reverse primer for protein expression |
| BAP2592 | CACCATGGCGACTATTGATTCAAAAACA | PM1501 - forward primer for protein expression |
| BAP2593 | ATCAATTTCATTTAAGATATCTTGAGCGAG | PM1501 - reverse primer for protein expression |
| BAP2614 | CACCATGAGCGGCGGTGGCGGTAGC | PM1517 - forward primer for protein expression |
| BAP2615 | TTGTGCTTGGTGACTTTTTTCAGC | PM1517 - reverse primer for protein expression |
| BAP2527 | CACCATGGATGACAAGAAGACCGCAACG | PM1578 - forward primer for protein expression |
| BAP2528 | TTTTTTACTTACTTCGTCATAATATTTTTGCGCAC | PM1578 - reverse primer for protein expression |
| BAP2808 | CACCATGCAACGAGAAGAAGACCCGGT | PM1611 - forward primer for protein expression |
| BAP2807 | CTCCCAATCACACCCTTCTG | PM1611 - reverse primer for protein expression |
| BAP2806 | CACCATGAGTTCAAATACGCCTGCGCC | PM1614 - forward primer for protein expression |
| BAP2805 | ACGTTTTGGCAAATAACGGGTTGGATC | PM1614 - reverse primer for protein expression |
| BAP2596 | CACCATGCAAGCTAATTTAGAGAAATCAAC | PM1622 - forward primer for protein expression |
| BAP2597 | AAACCGATATTCTAATCCTAAATGC | PM1622 - reverse primer for protein expression |
| BAP4325 | CACCATGATGGATCAACTTGAAATGAAAAAAAT | PM1670 - forward primer for protein expression |
| BAP4324 | TTCAATGACTTTGGCACCTTCAG | PM1670 - reverse primer for protein expression |
| BAP2804 | CACCATGGGTCAGTATCCAGATTTACCAGA | PM1707 - forward primer for protein expression |
| BAP2803 | TTCTACTGTTAATTTCTTACCATCATAAC | PM1707 - reverse primer for protein expression |
| BAP2802 | CACCATGTCCGTTAATACAACATCTTGTGGT | PM1717 - forward primer for protein expression |
| BAP2801 | AAACAGAATATTGAAACCAACATTAAGC | PM1717 - reverse primer for protein expression |
| BAP2529 | CACCATGTCCGGCTCTAAAGATGTTG | PM1720 - forward primer for protein expression |
| BAP2530 | TTGTTTTAATAAGCCTTCAGGCTCCG | PM1720 - reverse primer for protein expression |
| BAP2584 | CACCATGAAAGATGATAAACCGGCAGC | PM1730 - forward primer for protein expression |
| BAP2585 | CCAACCTTTAACTACACCACCTTG | PM1730 - reverse primer for protein expression |
| BAP2794 | CACCATGCAACAATTAGAACTACAAGTA | PM1809 - forward primer for protein expression |
| BAP2793 | AAGTTCCGCGCCTAGTCCA | PM1809 - reverse primer for protein expression |
| BAP2792 | CACCATGCAAAATACCGGGGGCAGT | PM1826 - forward primer for protein expression |
| BAP2791 | TCCTATTTGAATTGCAGAACGCTG | PM1826 - reverse primer for protein expression |
| BAP2788 | CACCATGTCAACTGTAGATAAGCTTGTGTATC | PM1886 - forward primer for protein expression |
| BAP2787 | TTGCCAAAATTTCCACCAGC | PM1886 - reverse primer for protein expression |
| BAP2784 | CACCATGAAAGGAAGCTACGGTATCGG | PM1898 - forward primer for protein expression |
| BAP2783 | AGAATGGCTAATAATTCGACTTCAAAC | PM1898 - reverse primer for protein expression |
| BAP4328 | CACCATGGCAGAGGCACTCGCTAAGCATATTAAA | PM1974 - forward primer for protein expression |
| BAP4329 | AAGCACTTTCGGTAAACGAATTTCCTG | PM1974 - reverse primer for protein expression |
| BAP2600 | CACCATGCAGCAGACCGAAGTGGATTTAC | PM1980 - forward primer for protein expression |
| BAP2601 | TTGCGATTGCCAACCAACG | PM1980 - reverse primer for protein expression |
| BAP2590 | CACCATGGCGCCGTTTGTAGTGAAAG | PM1992 - forward primer for protein expression |
| BAP2591 | GAACGTCCCACCAATGCTGA | PM1992 - reverse primer for protein expression |
| BAP2669 | CACCATGACAGAAAATATTGCATTTATCAGTG | PM1993 - forward primer for protein expression |
| BAP2668 | TTTCGCCTGTGTCGCAGGAATG | PM1993 - reverse primer for protein expression |
| BAP2671 | CACCATGTTTGTGTTAAGCGGGATTACA | PM1979 - forward primer for protein expression |
| BAP2670 | TTGCTCTTCATTTTGCTGGA | PM1979 - reverse primer for protein expression |
| BAP2667 | CACCATGGTGAGTCAAGAAGGTAACAAAAC | PM2008 - forward primer for protein expression |
| BAP2666 | CTCAATATGTGGCGGAAATTTTTTTGCTC | PM2008 - reverse primer for protein expression |
